# Individualized Selection of Valve Intervention Strategies in Aortic Disease Is Key for Better Outcomes

**DOI:** 10.3390/jpm15080337

**Published:** 2025-08-01

**Authors:** Vasiliki Androutsopoulou, Prokopis-Andreas Zotos, Andrew Xanthopoulos, Evangelos Boultadakis, Dimitrios Magouliotis, Nikolaos Schizas, Dimitrios C. Iliopoulos, John Skoularigis, Thanos Athanasiou

**Affiliations:** 1 Department of Cardiothoracic Surgery, University Hospital of Larissa, 41110 Larissa, Greece; zotospro@hotmail.com (P.-A.Z.); vboultadakis@yahoo.gr (E.B.); t.athanasiou@imperial.ac.uk (T.A.); 2 Department of Cardiology, University Hospital of Larissa, 41110 Larissa, Greece; anxanthopoulos@med.uth.gr (A.X.); iskoular@med.uth.gr (J.S.); 3 Department of Cardiac Surgery Research, Lankenau Institute for Medical Research, Wynnewood, PA 19096, USA; dimitrios.magouliotis.18@alumni.ucl.ac.uk; 4 4th Cardiac Surgery Department, Hygeia Hospital, 15123 Athens, Greece; nikschizas@gmail.com (N.S.); diliopoulos@hygeia.gr (D.C.I.)

**Keywords:** valve intervention, aortic valve disease, mechanical prosthesis, aortic valve replacement, bio prosthesis, transcatheter valve

## Abstract

Aortic valve diseases affect a significant percentage of the population, and with the extension of survival expectancy, they are expected to increase furthermore. Surgical treatment of aortic valve diseases mainly includes valve replacement and, rarely, its repair. The technology of both surgical and transcatheter valves is evolving, and new prosthetic valves with improved characteristics are available, e.g., longer lifespan, faster implantation, better hemodynamic performance with better effective orifice area, suitable for small aortic annuli, etc. Minimally invasive surgical techniques are constantly evolving and spreading. New access sites are used for transcatheter valve implantation. The Heart Team determines the most appropriate intervention for each patient based on their anatomical and clinical profiles, aiming to optimize long-term outcomes.

## 1. Introduction

Aortic valve diseases affect a significant percentage of the population worldwide and are expected to increase further with the extension of life expectancy and the aging of the population. Aortic valve stenotic disease is the most commonly occurring valvular pathology, striking nine million people globally. It is highly age-related and the most common cardiovascular disease in high-income countries. Aortic valve insufficiency has been increased in the developed world and ranks fourth worldwide [1,2].

Aortic valve diseases cause decreased exercise capacity, life expectancy, and quality of life. Diseases of the aortic valve are responsible for 61% of all valvular heart disease deaths.

Aortic valve replacement (AVR) is the most common form of valve surgery worldwide. Replacement options include bioprosthetic valves, mechanical valves, aortic valve homografts, and pulmonary autografts. The optimal selection of valve intervention in aortic valve disease is still in a focus of interest, and it is evolving rapidly. Personalized treatment of aortic valve diseases is associated with reduced postoperative complications, improved long-term outcomes, and enhanced quality of life.

This review presents the latest developments, outcomes, and concerns of aortic valve interventions.

## 2. Indications for Aortic Valve Surgery

According to the current 2021 ESC/EACTS Guidelines for the management of valvular heart disease, aortic valve replacement (AVR) has a Class I recommendation for symptomatic patients with severe high-gradient aortic stenosis and for symptomatic patients with low-flow, low-gradient aortic stenosis with reduced ejection fraction and evidence of flow reserve.

In asymptomatic patients with severe aortic stenosis and left ventricular dysfunction or symptoms on exercise testing, the recommendation is also Class I. Likewise, the recommendation for patients with severe aortic stenosis undergoing another cardiac surgery is Class I.

The recommendation for aortic valve replacement in patients with severe symptomatic aortic valve insufficiency and in patients with severe asymptomatic insufficiency with LVESD > 50 mm or resting LVEF ≤ 50% is Class Ι.

The recommendation for patients with severe aortic insufficiency regardless of symptoms, undergoing another cardiac surgery is Class I [3].

## 3. Criteria for Choosing Surgical Mechanical or Bioprosthetic Valve

The choice of biological or mechanical valve is a multifactorial process, influenced by the age of the patient and many other parameters. Factors for valve selection are bleeding and thromboembolic risks related to anticoagulation, the potential need for and risk associated with future reintervention, comorbidities, and the patient’s preference. Equally important parameters are the patient’s life expectancy and lifestyle, the valve’s durability, and the risk of the valve’s early degeneration.

Mechanical prostheses are durable and associated with fewer reinterventions, but they are also thrombogenic and require lifelong anticoagulation, exposing patients to thromboembolic and hemorrhagic complications. These complications have contributed to the significant increase in bioprosthetic valve implantations worldwide [4].

According to the 2021 ESC/EACTS Guidelines for the management of valvular heart disease, the desire for a valve type of a well-informed patient is a Class I recommendation (Level C). A bioprosthetic valve is also recommended (Class I, Level of Evidence C) when good-quality anticoagulation is unlikely or contraindicated due to high bleeding risk, when a patient’s life expectancy is lower than the presumed durability of the prosthesis, and finally in the case of reoperation for mechanical valve thrombosis despite good anticoagulation control.

An aortic bioprosthesis should be considered Class II a, Level of Evidence C in patients with low likelihood or low periprocedural risk for future reinterventions, in women contemplating pregnancy, and in patients older than 65 years old.

An aortic mechanical prosthesis is recommended (Class I, Level of Evidence C) in patients at high risk of accelerated structural valve degeneration. An aortic mechanical prosthesis should be considered (Class II a, Level of Evidence C) in patients aged less than 60 years old, in patients with reasonable life expectancy and high periprocedural risk for future reinterventions, or in patients with another mechanical prosthetic valve in other position [3].

Between the European and the American guidelines for the age limit for choosing a mechanical or bioprosthetic aortic valve, there is no consensus.

According to the 2020 ACC/AHA Guideline for the Management of Patients with Valvular Heart Disease, it is reasonable (Class II a, Level of Evidence B) to choose a mechanical aortic prosthesis for patients aged less than 50 years old without contraindication to anticoagulation or to choose an aortic bioprosthesis for patients older than 65 years old. It is also reasonable (Class II a, Level of Evidence B) for patients from 50 to 65 years old without contraindication to anticoagulation to customize the valve selection with consideration of patient factors and after informed decision-making [5].

There is a decrease in mechanical valve implantations in younger patients and a corresponding increase in surgical bioprosthetic valve implantations. In the US, there has been an increase in biological valve implantations in the aortic position from 14% in 1997 to 47% in 2014 [6]. Similarly, in a large European study, a three times increase in the number of bioprosthetic valve implantations in the aortic position was found in 2016 compared to 2006 [7].

Biological valves are now preferred in younger patients, despite their higher rates of early degeneration. More patients wish to avoid the potential complications of long-term anticoagulant therapy, dietary restrictions, drug interactions of anticoagulants, frequent blood tests, and restrictions on sports activities. The advanced technology of newer-generation biological valves—on the one hand, with improved maintenance conditions before implantation, longer life, better hemodynamic profile or expandable annulus, and the alternative of a percutaneous valve-in-valve implantation when the original bioprosthetic valve degenerates—have played a significant role in the increase in bioprosthetic valve implantations [7,8,9]. The special characteristics of the different types of bioprosthetic aortic valves are illustrated in the following table (Table 1).

## 4. Aortic Homografts

Aortic homografts are human biological valve conduits with excellent hemodynamic parameters and excellent anti-infective properties. Their main disadvantage is that they have higher rates of structural valve degeneration, compared to aortic bioprostheses.

In adults, homografts are generally reserved for aortic valve endocarditis with or without aortic root abscess, due to their resistance to infection.

The implantation of the homograft is technically demanding, and the risk of early structural valve deterioration is high. The risk of reoperation is also high. Decellularized aortic homografts seem to be a better alternative than the cryopreserved ones. Neither of the homografts need lifelong anticoagulation [10,11].

## 5. The Ross Procedure

The Ross procedure is a more complicated and technically demanding procedure than standard aortic valve replacement. During the procedure, the patient’s pulmonary autograft replaces the diseased aortic valve. It is an attractive option when VKA anticoagulation is contraindicated or undesirable, especially for young and middle-aged adults and for women contemplating pregnancy.

The Ross procedure is associated with better long-term outcomes and superior hemodynamic performance compared with conventional aortic valve replacement. In fact, in appropriately selected patients and in high-volume centers, this procedure improves long-term survival.

The potential failure of both the aortic and the pulmonary valve has been considered the main disadvantage of the Ross procedure [12].

## 6. New-Generation Surgical Aortic Valves

The bioprosthetic surgical aortic valves are constantly evolving, with innovations in their design, features, improved durability, and better hemodynamic performance.

The rapid deployment (Intuity Elite) and the sutureless aortic bioprosthetic valves (Perceval S) give the advantage of shorter aortic cross-clamp time and shorter extracorporeal circulation time and are probably associated with fewer postoperative complications compared to classic bioprosthetic valves. They are recommended in elderly, frail patients with comorbidities and high perioperative risk. These valves are also used in combination with minimally invasive surgical techniques. It should be noted that they are associated with more permanent pacemaker implantations. Large, randomized studies are expected, which will confirm their long-term results and hemodynamic behavior [13,14,15].

The bioprosthetic pericardial aortic valves (Inspiris Resilia) with an expandable annulus have the advantage of allowing a transcatheter valve-in-valve implantation of a larger valve size after their degeneration, to avoid patient–prosthesis mismatch. They are designed to deliver a controlled and predictable expansion during valve-in-valve deployment. In addition, due to a modified manufacturing process, they promise a longer lifespan with less calcium deposits in their cusps. The COMMENCE trial showed excellent 7-year results for RESILIA technology in terms of their hemodynamic behavior, the absence of insufficiency, and the absence of signs of early degeneration. The Inspiris Resilia valve is indicated in patients who are indicated for biological valve implantation but have a longer life expectancy [16,17,18].

The Foldax^®^ Tria™ valve is a new polymer-based prosthetic valve, robotically produced with resistance to calcification. It is computer-designed and offers large effective orifice areas and excellent hemodynamics. It is expected to have great durability and biocompatibility, without requiring anticoagulants. TRIA valve has been studied in clinical trials in the US and approved by the FDA, but it is not available for commercial sale at the moment. It is a promising alternative to overcome the current limitations of bioprosthetic and mechanical valves [19,20].

Tissue-engineered heart valves are experimental valves mainly fabricated from decellularized xenogeneic heart valves with repair, remodeling, and regeneration capabilities. In fact, these valves have the potential to overcome the limitations of prosthetic surgical valves as they will be scaffolds for the in situ generation of native-like autologous valves. These biocompatible valves will be valuable, especially in younger patients with valvular heart diseases. Heart valve tissue engineering still has serious challenges to overcome, such as thrombosis and immune response, which need to be further studied [21,22,23].

## 7. Minimally Invasive Aortic Valve Replacement

Minimally invasive aortic valve replacement techniques are now used worldwide. It is a safe alternative to conventional aortic valve replacement. Observational studies have shown that they cause less trauma, less transfusions, less hospital stay, less ICU stay, less pain. They also provide better cosmetic outcomes, lower risk of wound infections and faster return to daily activities. The disadvantages of the method include greater learning curve, greater aortic cross-clamp, CPB times and the need for expensive equipment.

Minimally invasive aortic valve replacement is performed via mini sternotomy or via right anterior thoracotomy. The combination of minimally invasive surgical techniques and sutureless or rapid deployment aortic valves has significantly reduced the operative time, which is especially important in elderly and high-risk patients [24,25,26].

There are a few randomized controlled trials assessing the efficacy and risks of minimally invasive aortic valve replacement compared with conventional aortic valve replacement. The small size of the sample and the incomplete collection of postoperative outcomes, reduce the trial’s power. According to a randomized trial, minimally invasive aortic valve replacement did not cause less bleeding compared to conventional sternotomy [27]. Another randomized trial concludes that aortic valve replacement through upper hemisternotomy did not reduce red blood cells transfusion during the first postoperative week [28]. According to a third randomized trial, there is no significant difference between mini-sternotomy and full sternotomy aortic valve replacement in terms of all-cause mortality, rate of reintervention, major adverse cardiac and cerebrovascular events and echocardiographic data after 6.1-year follow-up [29]. Therefore, larger randomized trials are required for reliable results on the real benefits of minimally invasive aortic valve replacement.

## 8. Small Aortic Annulus

The small aortic annulus is a serious surgical challenge in patients undergoing aortic valve replacement. To avoid patient–prosthesis mismatch there are many management options like aortic root enlargement, aortic root replacement and the use of sutureless and stentless bioprosthetic valves. Aortic root enlargement and replacement procedures are time-consuming and technically demanding, leading to prolonged cross-clamp and cardiopulmonary bypass times.

Sutureless and stentless aortic bioprosthetic valves allow the surgeon to avoid complicated aortic root procedures. They effectively prevent patient–prosthesis mismatch and offer the advantage of a larger effective orifice area. The self-anchoring delivery system of sutureless valves reduces cardiopulmonary bypass and cross-clamp times [18,30,31,32].

## 9. Bicuspid Aortic Valve

Bicuspid aortic valve is the most common congenital heart disease. It is associated with high rates of calcific aortic valve stenosis, aortic insufficiency, aortic root, and ascending aorta dilatation. Bicuspid aortic valve usually requires intervention a decade earlier than the tricuspid valve. Up to 50% of individuals with a bicuspid aortic valve will have an aortic valve replacement in their lifetime.

According to the 2020 ACC/AHA and the 2021 ESC/EACTS guidelines for the management of valvular heart disease, surgical aortic valve replacement is the gold-standard treatment for patients with severe aortic stenosis or pure regurgitation of a bicuspid valve [3,5].

Surgery remains the treatment of choice, especially in younger low-risk patients and in patients with concomitant aortic dilatation (Table 2).

Surgery for bicuspid aortopathy in patients undergoing aortic valve surgery should be considered at an aortic root or ascending aorta diameter more than 4.5 cm [33,34,35].

**Figure 1:**
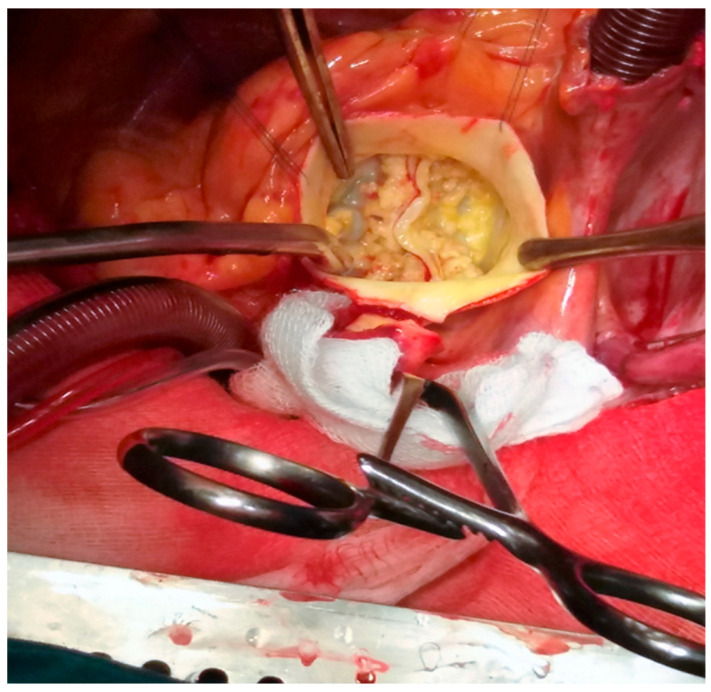
Intraoperative picture of a bicuspid valve in a patient with concomitant aortopathy (personal collection).

Surgical aortic valve replacement has low in-hospital mortality, ranging from 0.9% to 2.4%, and 10-year survival greater than 80%. The surgical procedure has lower rates of pacemaker implantation and paravalvular leak than transcatheter valve implantation (Figure 1).

Rapid deployment and sutureless valves are usually avoided in patients with bicuspid valves due to the higher risk of paravalvular leak and pacemaker implantation [36].

Patients with bicuspid aortic valve are usually excluded from large randomized controlled TAVI trials. Nevertheless, 5–10% of patients undergoing TAVI have bicuspid aortic valve. The significant leaflet and annular calcification in bicuspid aortic stenosis, in conjunction with the concomitant aortopathy, increases the risk of pacemaker implantation, annular rupture, and paravalvular regurgitation after TAVI implantation. Bicuspid aortic valve patients have a higher risk of transcatheter valve underexpansion and increased risk of leaflet thickening and valve thrombosis [37,38]. According to the data from the NOTION 2 trial, low-risk patients younger than 75 years old with bicuspid aortic valve, treated with transcatheter aortic valve implantation, have higher rates of moderate paravalvular regurgitation, death, and non-disabled stroke [39].

## 10. Aortic Valve Endocarditis

Infective endocarditis is a life-threatening complication affecting either the native aortic valve or an aortic prosthesis. The reported incidence of endocarditis, following transcatheter aortic valve replacement, is 0.3–2.0 per 100 person-years. Surgical aortic valve endocarditis affects approximately 3–10 per 1000 person-years. The risk may be even higher in elderly patients with multiple rehospitalizations and those undergoing invasive procedures such as hemodialysis. The most common complications of prosthetic valve endocarditis are valve disfunction, septic shock, acute kidney and heart failure, and systemic embolism. Prosthetic valve endocarditis is associated with a higher risk of in-hospital mortality [40,41].

Whenever surgical treatment is indicated in native and prosthetic aortic valve endocarditis, surgical aortic valve replacement is usually required, and the use of an allograft is still preferred (Table 2). It is well known that bioprostheses are associated with lower reinfection rates compared to mechanical valves. In children and middle-aged patients in high-volume centers, the Ross operation is usually indicated. If there are no other contraindications, patient preferences are considered. The implantation of transcatheter aortic valve in endocarditis is contraindicated. In native valve endocarditis, valve repair is performed whenever it is feasible [42].

## 11. Transcatheter Aortic Valve Implantation

Over the last two decades, transcatheter aortic valve implantation technology has rapidly evolved. With advances in procedural techniques and device technology, transcatheter aortic valve replacement has become a safe alternative to surgical aortic valve replacement in selected patients with severe symptomatic aortic valve stenosis (Table 2). New commercially available transcatheter aortic valves, especially designed to treat native aortic insufficiency, have shown satisfying short-term outcomes. Also, newly designed valves with smaller delivery systems (e.g., 14F catheters) allow valve implantation without surgical incision and eventually with less vascular complications. Transcatheter aortic valve repair for pure aortic regurgitation will be feasible in the future with devices like the Cusper device, which is designed to fill the effective regurgitant orifice of the valve [43].

Over the years, the TAVI procedure has become less invasive. The minimalistic TAVI approach includes local anesthesia and conscious sedation instead of general anesthesia, percutaneous vascular access instead of surgical cut-down, percutaneous femoral artery closure, and the radial artery as secondary access. This approach is associated with reduced procedural time, superior outcomes, a shorter length of hospital stay, and a decrease in post-procedural complication rates [44].

The Heart Team takes into consideration patient’s preference, clinical and anatomical characteristics, procedural factors, and experience in order to select the optimal treatment. Factors that favor transcatheter aortic valve implantation are older age, prior chest radiation, higher surgical risk, previous cardiac surgery with intact coronary artery bypass grafts, porcelain aorta, frailty, short life expectancy, and severe chest deformity. Transcatheter aortic valve implantation is now approved for younger (less than 70 years old) and lower-risk patients, supported by long term outcomes from the Partner 3 and Evolut Low Risk Trials.

There is no complete agreement between the 2021 ESC/EACTS Guidelines and the 2020 ACC/AHA Guideline on the selection criteria for transcatheter valves [3]. According to the 2021 ESC/EACTS Guidelines for the management of valvular heart disease, transcatheter aortic valve intervention is recommended in patients older than 75 years old or in those who are high risk (STS score > 8%) or unsuitable for surgery (Class I, Level of Evidence A). On the other hand, surgical aortic valve replacement is indicated in patients younger than 75 years old who are at low risk for surgery (STS score < 4%), in patients unsuitable for transfemoral TAVI (Class I, Level of Evidence B), or in patients with severe aortic stenosis undergoing another cardiac surgery (Class I, Level of Evidence C) [3].

According to the 2020 ACC/AHA Guideline for the management of patients with valvular heart disease, in severe symptomatic or asymptomatic aortic stenosis, surgical aortic valve replacement is recommended in patients younger than 65 years old or in patients with a life expectancy of more than 20 years (Class I, Level of Evidence A). In symptomatic severe aortic stenosis, transcatheter aortic valve intervention is recommended in patients older than 80 years old or in patients with a life expectancy less than 10 years, with no anatomic contraindications to transfemoral TAVI (Class I, Level of Evidence A). For patients aged between 65 and 80 years old with severe symptomatic aortic stenosis and no anatomic contraindications to transfemoral TAVI, either surgical or transcatheter aortic valve replacement is recommended (Class I, Level of Evidence A) [5].

Cardiac computed tomography plays a crucial role in the pre-procedural planning of transcatheter aortic valve implantation, providing accurate assessments of the native aortic valve morphology and aortic root and annulus dimensions. CT angiography also identifies the location of the coronary artery ostia and measures their distance from the aortic annulus. High-resolution imaging ensures accurate valve sizing and appropriate valve type selection. These measurements are also valuable for anticipating uncommon complications, such as elongated aortic valve leaflets that could increase the risk of coronary artery occlusion during valve deployment [45].

Paravalvular leak (PVL) represents a significant complication following transcatheter aortic valve implantation, with notable adverse effects on patient outcomes. The reported incidence of PVL in various studies ranges from 7% to 40%. Evidence suggests that PVL severity is typically highest at implantation and tends to decrease over time. Risk factors for paravalvular leak following TAVI include increased aortic root calcification volume, larger annulus dimensions, elevated pre-TAVI transvalvular peak velocity, as well as undersizing, underexpansion, or malpositioning of the prosthesis.

VARC-3 criteria are essential for precise PVL assessment after TAVI. Moderate to severe PVL raises all-cause mortality by two- to three-fold and can cause heart failure symptoms, kidney injury, early prosthetic valve deterioration, and an increased need for aortic reintervention. A reduced incidence of PVL has been observed following the implantation of new-generation transcatheter aortic valves [46].

The reported incidence of paravalvular leak following surgical aortic valve replacement varies between 1% and 10%, according to multiple studies.

Permanent pacemaker implantation is an established complication associated with transcatheter aortic valve implantation (TAVI). The incidence of pacemaker implantation after TAVI is approximately 19%, with slightly higher rates observed in first-generation TAVI prostheses and self-expandable valves. Post-TAVI pacemaker implantation has been linked to an increased risk of all-cause mortality and rehospitalization for heart failure.

Risk factors for permanent pacemaker implantation include patient characteristics (such as older age and male sex), baseline ECG findings (including RBBB, LBBB, and first-degree atrioventricular block), procedural aspects (such as valve oversizing and implantation depth), and the type of valve used (self-expandable or first-generation valve). Common patient-associated risks include a short membranous septum, a small or eccentric left ventricular outflow tract (LVOT) with large annuli, and increased calcium volume below the non-coronary cusp.

In general, balloon-expandable valves and newer-generation prostheses have been associated with lower rates of pacemaker implantation. Valves with a diameter of 29 mm or greater are associated with a higher risk of pacemaker implantation [47,48].

Acute ischemic stroke continues to be a significant complication associated with transcatheter aortic valve replacement. Approximately half of all neurological events occur during the periprocedural period, with about 50% arising within the first 24 h. These events are primarily attributed to the cerebral embolization of debris or thrombus, as well as hypoperfusion. Major stroke following TAVR is linked to a greater than fivefold increase in one-year mortality.

Cerebral embolic protection devices were designed to reduce the risk of acute ischemic stroke associated with transcatheter aortic valve replacement. However, current evidence does not definitively demonstrate the efficacy of these devices in preventing strokes [49,50].

The surgical cut-down approach has been reported to have a lower incidence of vascular complications, whereas the percutaneous approach is considered a less invasive method. With enhanced patient preprocedural screening, greater operator experience, smaller sheaths, and advancements in device technology, the percutaneous approach has become more efficient. Percutaneous vascular closing techniques and devices have been increasingly utilized in clinical practice.

To avoid vascular complications, the common femoral artery puncture site should be identified on CT or angiography before the procedure, with the puncture performed under angiographic or ultrasound guidance.

Using the radial artery as secondary access, rather than the contralateral femoral site, may significantly reduce vascular complications [44].

Coronary obstruction is an uncommon but fatal complication of transcatheter aortic valve implantation, with an incidence ranging from 0.5% to 8%. This condition carries a high risk, as the reported in-hospital 30-day mortality rate is from approximately 30% to 50%.

Risk factors for coronary obstruction during transcatheter aortic valve implantation include low coronary ostial height, narrow sinuses of Valsalva, small aortic annulus, bicuspid aortic valve, valve-in-valve procedure, balloon-expandable valve implantation, and valve oversizing. The BASILICA procedure can be used to prevent coronary ostia obstruction by puncturing leaflets [51].

The expansion of transcatheter aortic valve replacement to younger and lower-risk patients with longer life expectancy has raised questions about long-term valve durability, valve thrombosis, coronary access, conduction abnormalities requiring pacemaker implantation, paravalvular leak, stroke, and the likelihood of valve reintervention.

Hypoattenuated leaflet thickening (HALT), is a possible complication after transcatheter aortic valve implantation that may affect valve hemodynamic performance and long-term outcomes. Recent studies, using functional cardiac computed tomography, have shown early valve thrombosis at the base of prosthetic aortic leaflets. HALT is associated with early prosthetic valve degeneration and a high probability of thromboembolic neurological and cardiac events. According to an observational study, HALT was found in 12% of patients the first post-procedural month after TAVI. Warfarin therapy was effective in 80% of patients with HALT [52,53].

The transfemoral approach has been the preferred approach for transcatheter aortic valve implantation, performed either percutaneously or by surgical exposure. This approach, in appropriate femoral artery anatomy is efficient, safe, and usually does not require general anesthesia. Vascular complications in transfemoral implantation are rare, but they can be catastrophic.

For patients with severely calcified, small-diameter, tortuous iliofemoral arteries or significant descending aortic pathology, the transfemoral access is not recommended. In such circumstances, there are many alternative access options like transcarotid, transaxillary, transaortic, transapical, and transcaval. Transapical and transaortic accesses require general anesthesia and sternotomy or thoracotomy. Transapical access tends to be abounded because of the greater perioperative risk. Transcarotid access is the most used when transfemoral access is not feasible. Transcaval access avoids manipulation of the head and neck vessels but is associated with higher rates of vascular complications [54].

There are currently many commercially available transcatheter aortic valves: the Sapien Edwards balloon-expandable valve, the Medtronic Evolut self-expanding valve, the St. Jude Portico self-expanding prosthesis, and the Abbott Navitor self-expanding valve with their delivery systems. The JenaValve prosthesis is the first transcatheter valve to receive a CE mark for the treatment of both aortic regurgitation and aortic stenosis. EvolutFX Medronic is a new generation self-expandable transcatheter aortic valve designed to provide greater precision and control throughout the implantation procedure. The system was designed to improve delivery ability and assess commissural alignment [55]. The new SAPIEN 3 Ultra RESILIA balloon-expandable transcatheter aortic valve is treated with special calcium-blocking tissue technology, the same used in the surgical RESILIA INSPIRIS valve, with potential improved durability and reduced risk of reintervention. According to the data of the Ocean TAVI Registry, the SAPIEN 3 Ultra RESILIA valve has equivalent in-hospital death, vascular complications, and new pacemaker implantation but better hemodynamic performance and less paravalvular leakage than the Sapien 3 valve [56]. Self-expanding transcatheter aortic valves provide a larger effective orifice area. Better hemodynamic performance and better durability, but they are associated with a greater risk for conduction abnormalities and pacemaker implantation (Table 3).

On the other hand, balloon-expandable valves are associated with better positioning and deployment, better coronary access, and lower risk of pacemaker implantation [57,58].

## 12. The Structural Degeneration of Bioprosthetic Valves

The Achille’s heel of bioprosthetic valves remains their shorter lifespan and the need for reoperation. Structural valve deterioration (SVD) usually begins within the first five years of implantation and worsens after the decade. Its etiology is multifactorial. Patients younger than 50 years of age at implantation are more likely to develop valve degeneration.

According to a recently published series, subclinical degeneration of bioprosthetic surgical valves in patients younger than 65 years of age occurs in 43.3% of patients, with 21% of them presenting with clinically significant degeneration and leading to reoperation. The predicted risk of bioprosthetic valve degeneration at 15 years is 50% in patients aged 20 years at implantation, 30% in patients aged 40 years at implantation, and 22% in patients aged 50 years. The same risk in patients over 65 years is only 10% [59,60,61,62].

## 13. Management of Bioprosthetic Valve Degeneration

Surgical replacement of a degenerated bioprosthetic valve is the method of first choice, especially in young patients with low surgical risk; it is a safe option, with very good long-term results [52]. Alternatively, in elderly, frail, and high-perioperative-risk patients, transcatheter implantation, also known as valve-in-valve technique, can be applied. With this technique, the rates of patient–prosthesis mismatch are higher compared to classic surgical replacement. Overall, 25% of patients after the valve-in-valve technique present with residual stenosis and increased transvalvular pressure gradient. When the size of the degenerated bioprosthetic valve is less than or equal to 21, then 40% of patients will develop a transvalvular pressure gradient greater than 20 mmHg [63,64].

It is important to note that surgical valve ‘True ID’ may be smaller than the labeled valve size. The exact dimensions of a failed bioprosthesis must be calculated accurately by CT, MRI, or TEE, so that the appropriate transcatheter aortic valve size can be implanted. In order to reduce the patient–prosthesis mismatch after the valve-in-valve technique, especially in small sizes of bioprosthetic surgical valves, the bioprosthetic valve fracturing (BVF) technique is applied. Mechanical dilation of the pre-existing bioprosthetic valve is attempted with a high-pressure balloon. Not all surgical valves respond equally to this technique. The valves that respond best to BVF are the following: Epic, Mosaic, Magna, Magna ease, and the newer-generation Perimount valves [64,65,66].

The bioprosthetic valves with expandable annuli (Inspiris Resilia) for the aortic position were designed to prevent patient–prosthesis mismatch in future implementation of the valve-in-valve technique. These valves allow for controlled expansion of the valve annulus to accommodate a larger transcatheter valve [67].

Although the valve-in-valve technique is less invasive compared to surgery, it has higher rates of malposition and coronary obstruction. According to Vivid Registry data, the first-year mortality of patients with small surgical valves (label size < or equal to 21) after VIV was 25.2%, while that of intermediate sizes was 18.2%. In addition, after VIV TAVI, coronary access may be challenging, especially in small aortic root with low coronary ostia and in supraannular degenerative surgical valves. ViV-TAVI offers an excellent alternative to treat patients with failed bioprostheses; however, redo SAVR is associated with better survival over time and seems to be a protective factor for all-cause mortality 6 months after the procedure [59,60,61,62]. Severe patient–prosthesis mismatch is associated with more hospital readmissions due to heart failure, reduced remodeling of left ventricular hypertrophy, impaired renal function, reduced survival, and increased mortality. ViV TAVI was associated with higher 30-day and 6-month all-cause readmission [65,66,67,68,69,70,71,72].

While transcatheter aortic valves are implanted to younger and lower-risk patients, an increase in surgical transcatheter aortic valve explants is expected. Explanting a failing transcatheter aortic valve is a rising and complex operation due to potential injury to the aortic root, ascending aorta, mitral valve, or membranous septum. In the United States of America, this is the fastest-growing cardiac surgery procedure. Explanting a TAVI device to implant a new surgical valve is a technically demanding operation with higher mortality and morbidity compared to redo surgical prosthetic valve replacement [73]. Valve-in-valve is a safe alternative for degenerated aortic bioprostheses in older and frail patients with high operative risk. Redo surgical aortic valve replacement may be the preferred choice for younger patients with lower surgical risk, particularly those with small-sized surgical bioprostheses [74].

## 14. Aortic Valve Repair

The aortic valve replacement in patients with severe aortic insufficiency used to be the gold standard for the last decades (Table 2). Recently, safe repair techniques have been developed, which offer satisfying long-term results and relieve patients from the effects of prosthetic valve implantation (thromboembolic and hemorrhagic complications, bioprosthetic valve degeneration, etc.). The choice of surgical technique depends on the aortic valve pathology, the specific anatomical features of the patient’s aortic root, and the surgeon’s preference and experience. In some cases, it may include suturing of the cusp fenestration with or without pericardial patch implantation, removing the calcium deposits off the leaflets, central cusp plication, and annuloplasty [75]. The aortic valve repair is a safe and reproducible alternative, but the risk of recurrence is possible.

## 15. Innovative Therapies and Research

While surgical procedures for the treatment of aortic valve disease have made great progress in the last decades, pharmacological interventions are still lacking. It is well known that calcific aortic stenosis is associated with Lipoprotein(a), a low-density lipoprotein, and PCSK9. Several clinical studies have used statin therapy in order to delay the progression of aortic stenosis, like the SALTIRE and the ASTRONOMER trials, but unfortunately, they have not demonstrated any therapeutic benefit. New clinical trials using oligonucleotides to suppress the expression of Lipoprotein(a), or monoclonal antibodies to inhibit the expression of PCSK9, are showing promising outcomes [76,77]. Additionally, fibrin-targeted immunotherapy has shown limited efficacy in the treatment of calcific aortic valve disease [78]. Mutations in LPA and NOTCH genes seem to play a major role in the development of calcific aortic valve disease. Gene therapies are investigated for those pathways to halt calcification [79].

## 16. Discussion

Aortic valve replacement is the most common form of valve surgery worldwide. The ideal heart valve prosthesis is not yet available. Replacement options include mechanical and bioprosthetic surgical valves, transcatheter bioprosthetic aortic valves, homografts, and pulmonary autografts. The optimal selection of valve intervention in aortic valve disease is still in the focus of interest. It is widely recognized that patient care should be personalized. The Heart Team carefully assesses each patient’s unique characteristics, including age, comorbidities and preferences, procedural factors, and the institution’s expertise, to identify the most appropriate intervention with the aim of achieving the best possible long-term outcomes.

Over the last decade, the transcatheter aortic valve implantation technology has rapidly evolved. New transcatheter devices are now available with improved characteristics and better outcomes. Likewise, significant progress has been made in minimally invasive aortic valve surgery. New surgical bioprosthetic aortic valves are currently available with special characteristics for the treatment of older, high-risk, and frail patients and patients with a small aortic annulus [14,15].

The global trend is to increase the implantation of biological valves over time, despite their early degeneration and the greater need for reinterventions in order to avoid the thromboembolic and hemorrhagic complications of mechanical prosthetic valves. Younger patients who wish to receive a bioprosthetic aortic valve should be completely informed about the risks and the long-term outcomes of their choice [6,7].

The selection between a mechanical valve and a bioprosthetic should be multifactorial and patient-centered. The Heart Team should take into consideration patient’s clinical and anatomical characteristics, device-specific factors, procedural outcomes, local experience, and informed patient preference in order to select the best type of intervention [4].

In the real world, there is no complete agreement between the 2021 ESC/EACTS and 2020 ACC/AHA guidelines on the criteria for selecting a mechanical or bioprosthetic valve, as well as the criteria for selecting transcatheter versus surgical implantation. The result is that patients are treated differently on both sides of the Atlantic [3,5].

Several ongoing randomized controlled trials, such as Evolut Low Risk, SURTAVI, NOTION-2, PARTNER 3, and DEDICATE, are evaluating TAVR’s safety and effectiveness compared to SAVR in low- and intermediate-risk patients. Ongoing research aims to evaluate long-term outcomes, assess durability, and refine criteria for optimal valve selection in younger and lower-risk patients.

The Evolut Low Risk trial found that, at 5 years, patients with severe aortic stenosis who underwent either TAVR or surgery had similar rates of all-cause mortality or disabling stroke. Valve durability and performance were reported to be comparable in both groups [80].

The NOTION-2 trial compared TAVI and surgery in low-risk patients aged ≤75 years with severe symptomatic AS, including both tricuspid and bicuspid cases.

At 1 year, rates of death, stroke, or rehospitalization were similar for both groups. TAVI patients had lower risks of major bleeding and new-onset atrial fibrillation but higher risks of non-disabling stroke, permanent pacemaker implantation, and moderate or greater paravalvular regurgitation. According to this trial, outcomes for young bicuspid AS patients undergoing TAVI require further study [39].

The NOTION-2 trial also found that patients treated with self-expanding TAVR had similar 3-year all-cause mortality rates and a lower incidence of disabling stroke compared to those who underwent surgery. Valve performance was superior in the TAVR group [81].

In the 5-year PARTNER 3 trial follow-up, rates of death, stroke, or rehospitalization were similar for TAVR and surgery groups. Atrial fibrillation and bleeding were less common with TAVR, while paravalvular aortic regurgitation, valve thrombosis, and pacemaker implantation were less frequent with surgery [82].

In the DEDICATE trial, TAVI was noninferior to SAVR for 1-year mortality or stroke in low- or intermediate-risk severe aortic stenosis patients [83].

Further research is needed to assess late adverse events and evaluate the long-term durability and the optimal antithrombotic treatment of transcatheter heart valves, especially in young, low-risk patients with longer life expectancy [57,58].

The surgical replacement of a failing transcatheter aortic valve is a rising operation with increased periprocedural risk, requiring special technical skills and surgical experience. While TAVR is increasingly being performed in younger and lower-risk patients, an increase in surgical transcatheter aortic valve explants is expected. Currently, the surgical replacement of a failing transcatheter aortic valve is the fastest-growing cardiac surgery procedure in the United States. Despite this, there is an absence of guidelines for the proper surgical management of patients undergoing redo-TAVR [73].

The valve-in-valve technique offers a less invasive alternative to treat patients with failed aortic bioprostheses, but it has higher rates of valve malposition, coronary obstruction, and patient–prosthesis mismatch. The impact of valve-in-valve technique on long-term survival has not been yet resolved. Redo surgical aortic valve replacement is associated with better survival over time and seems to be a protective factor for all-cause mortality, 6 months after the procedure. Redo surgical aortic valve replacement seems to be the preferred choice for younger patients with low surgical risk, particularly those with small-sized surgical bioprosthesis [68,71].

Minimally invasive cardiac surgery in combination with newer-generation surgical aortic valves (sutureless and rapid deployment valves) may be the answer to rapidly developing transcatheter valve technology. Many observational trials show advantages of minimally invasive aortic valve replacement (less trauma, short recovery time, less pain, less transfusions, less time in hospital, and shorter intensive care unit stay), but the truth is that there is a lack of data from large randomized controlled trials. Many cardiac surgeons believe that the main advantage of minimally invasive aortic valve replacement is the cosmetic result [27,29].

Nowadays in elderly, frail, and high-risk patients, with severe aortic stenosis and concomitant cardiac diseases (e.g., severe mitral regurgitation), in order to reduce the periprocedural risk, a hybrid approach is indicated. Simultaneous transcatheter aortic valve implantation and conventional cardiac surgery (e.g., mitral valve repair), seem to be a safe alternative [84].

## 17. Conclusions

The Heart Team, by examining the different techniques and available devices for the treatment of aortic valve diseases, has the ability to provide personalized treatment for each patient, aiming for the best possible long-term results. The implementation of patient-specific interventions strategies enhances procedural success rates and reduces the risk of complications. This personalized approach in aortic valve disease is key for better outcomes.

## Figures and Tables

**Table 1:** Characteristics of bioprosthetic surgical aortic valves.

	Durability	Less CPB Time	Less Cross Clamp Time	Small Aortic Annulus	Minimally Invasive Surgery	Less PPM	Pacemaker Implantation	Designed for Future Valve in Valve Procedure
Sutureless Valves		√	√	√	√	√	√	
Rapid Deployment Valves		√	√		√		√	
Valves with Expandable Annulus	√							√
Stentless Valves				√		√		
Stented Valves	√							

**Table 2:** Indications for surgical vs transcatheter aortic valve intervention.

	Aortic Endocarditis	Bicuspid Aortic Valve	Low Risk Patients	Young Patients	Aortic Regurgitation	Aortic Stenosis
Surgical Intervention	√	√	√	√	√	√
Transcatheter Intervention						√

**Table 3:** The differences between self-expanding and balloon-expandable transcatheter aortic valves.

	Durability	Large EOA	Pacemaker Implantation	Better Positioning	Better Coronary Access	Better Hemodynamic Performance
Self-expanding	√	√	√			√
Balloon-Expandable				√	√	

## Data Availability

No new data were created or analyzed in this study. Data sharing is not applicable to this article.

## Abbreviations

The following abbreviations are used in this manuscript:

LVESD	Left ventricular end-systolic diameter
PPM	Prosthesis-patient mismatch
SAVR	Surgical aortic valve replacement
TAVR	Transcatheter aortic valve replacement
STS	Society of Thoracic Surgeons
AVR	Aortic valve replacement
ESC	European Society of Cardiology
EACTS	European Association for Cardio-Thoracic Surgery
LVEF	Left ventricular ejection fraction
ACC	American College of Cardiology
AHA	American Heart Association
BVF	Bioprosthetic valve fracturing
VIV	Valve-in-valve
TAVI	Transcatheter aortic valve implantation
HALT	Hypoattenuated leaflet thickening
CT	Computed tomography
MRI	Magnetic resonance imaging
TEE	Transesophageal echocardiography
